# Low-grade myofibroblastic sarcoma arising in fibroadenoma of the breast-A case report–

**DOI:** 10.1186/s13000-016-0480-8

**Published:** 2016-03-25

**Authors:** Na-Hye Myong, Jun-Won Min

**Affiliations:** Departments of Pathology, Dankook University College of Medicine, 119 Dandae-ro, Dongnam-gu, Cheonan-si, Chungnam 31116 Korea; Surgery, Dankook University College of Medicine, Cheonan, Korea

**Keywords:** Breast, Fibroadenoma, Myofibroblasts, Pericytes, Sarcoma

## Abstract

**Background:**

Myofibroblastic sarcoma or myofibrosarcoma is a malignant tumor of myofibroblasts and known to develop rarely in the breast, but its underlying lesion and tumor cell origin have never been reported yet.

**Case presentation:**

A 61-year-old female presented with a gradually growing breast mass with well-demarcated ovoid nodular shape. The tumor was histologically characterized by fascicular-growing spindle cell proliferation with large areas of hyalinized fibrosis and focally ductal epithelial remnants embedded in myxoid stroma, mimicking a fibroadenomatous lesion. It had frequent mitoses of 5–16/10 high-power fields, hemorrhagic necrosis, and focally pericapsular invasion. The spindle cells were diffusely immunoreactive for fibronectin, smooth muscle actin, and calponin, which suggest a myofibroblastic origin. Multiple irregularly thickened vessels with medial or pericytic cell proliferation were found to be merged with the intrinsic tumor cells. The tumor could be diagnosed low-grade myofibroblastic sarcoma arising in an old fibroadenoma.

**Conclusion:**

We report a case of a low-grade mammary myofibrosarcoma that showed a background lesion of fibroadenoma first in the worldwide literature and suggest the pericytes or medial muscle cells of the intratumoral vessels as the cell origin of the myofibroblastic sarcoma.

## Background

Myofibroblastic sarcoma or myofibrosarcoma (MFS) is a malignant tumor which is composed of or originated from myofibroblasts. It has been known to arise mainly at head and neck regions, although it could be found at the extremities, trunk, and retroperitoneum. Mammary myofibrosarcomas have been very rarely reported, with only about 10 cases in the worldwide literature [1–7]. The mammary myofibroblastic sarcomas in the literature have revealed predominantly low-grade cytomorphology rather than high-grade tumor mimicking malignant fibrous histiocytoma (MFH) in the primary lesions. Thus, the main differential diagnoses of low-grade myofibrosarcoma (LG-MFS) in the breast range from nodular fasciitis and myofibroblastoma to fibrosarcoma, leiomyosarcoma, and cystosarcoma phyllodes [7, 8]. However, there has never been reported about the background lesion or cell origin of myofibroblasts in the worldwide literature, because all of the reported cases reveal neither epithelial component nor morphologic evidence for the cell origin anywhere as in the present case. We report a case of mammary myofibrosarcoma showing the background lesion of fibroadenoma and the pericytic cell origin of myofibroblastic tumor cells first in the worldwide literature.

## Case presentation

**Fig. 1:**
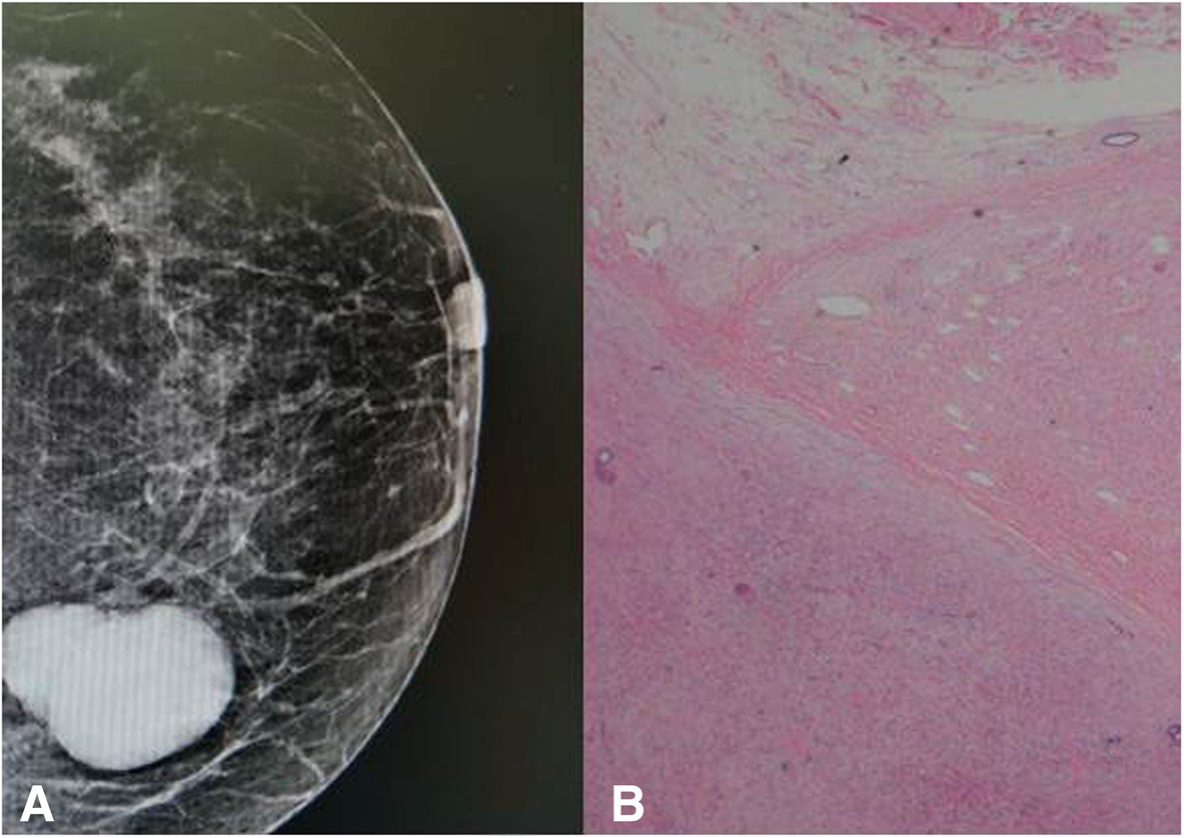
Mammographic finding and low-powered microscopic view of the breast mass. **a** A mammographic image revealed a well-circumscribed and highly dense nodular mass at 11 o’clock position. **b** The mass was well-circumscribed and mostly encapsulated, with the slightly lobulated margin (H&E stain, ×12.5)

**Fig. 2:**
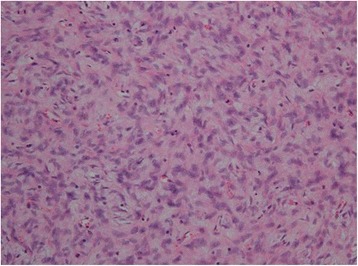
Representative histologic findings of the tumor. It is mostly composed of spindle-shaped mesenchymal cells with vaguely fasciculated growing pattern, elongated plump nuclei, pale eosinophilic cytoplasms, and small indistinct nucleoli (H&E stain, ×200)

**Fig. 3:**
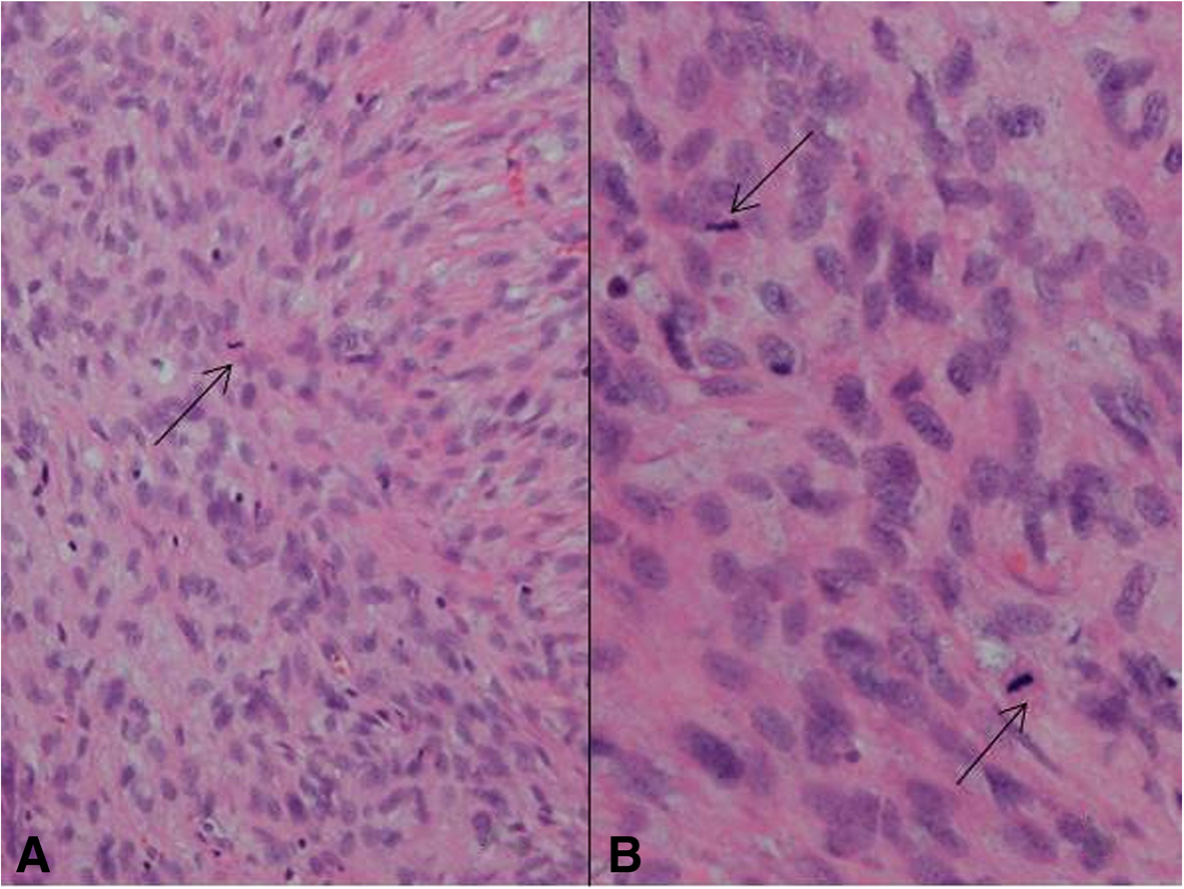
A cytologic finding suggestive of malignancy in this case. **a** The tumor cells show high cellularity and frequent mitotic figures (arrow) (H&E stain, ×200). **b** In one high-powered microscopic field, two mitotic figures (arrows) are observed (H&E, stain, ×400)

**Fig. 4:**
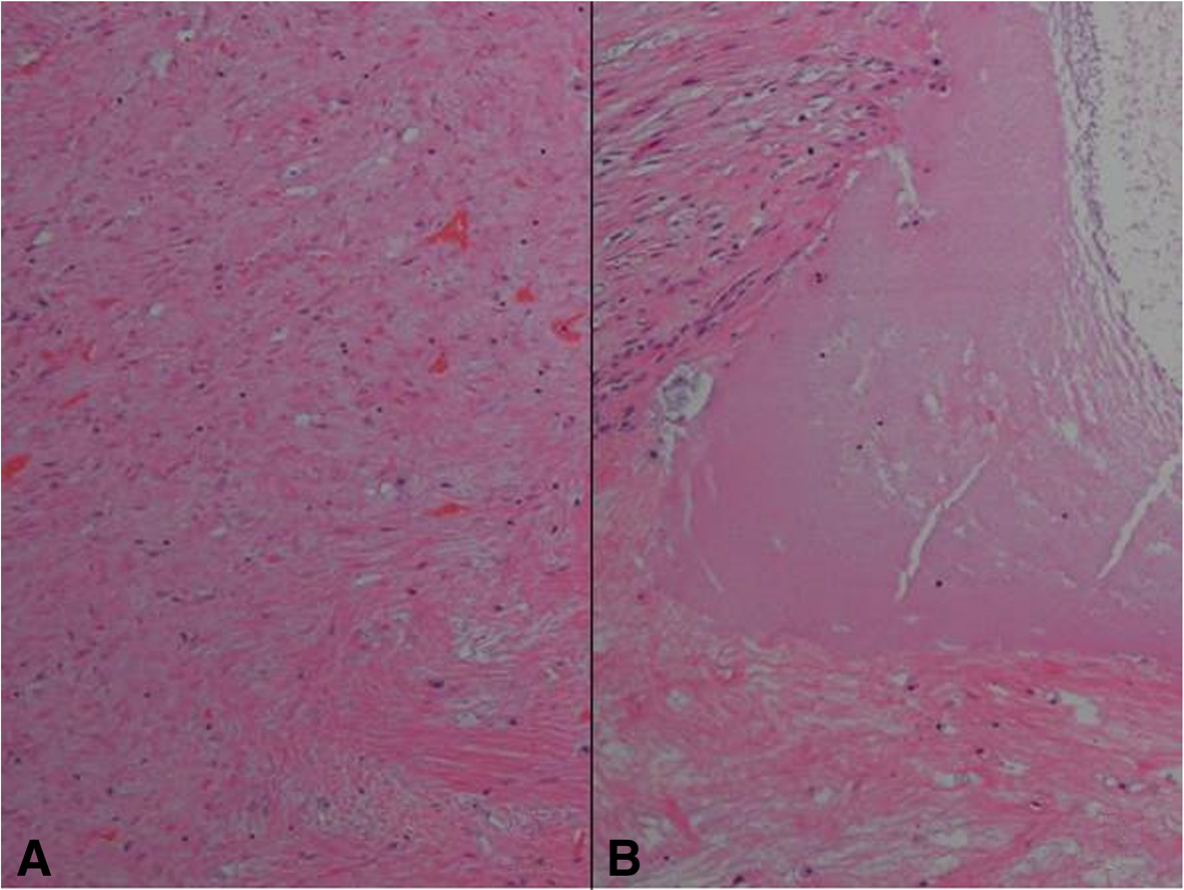
Two histologic findings indicative of degeneration in the tumor cells. **a** The tumor had focally hemorrhagic necrosis in about 5 % of the entire mass (H&E stain, ×200). **b** There was a few scattered foci of pseudocystic degeneration (H&E stain, ×200)

**Fig. 5:**
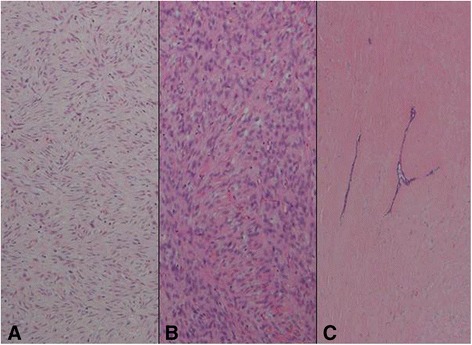
Variable stromal backgrounds according to the tumor cellularity. **a** Myxoid stroma is representatively found in moderately cellular tumor areas (H&E stain, ×100). **b** Slightly fibrous stromal background is diffusely noticed in the highly cellular areas (H&E stain, ×100). **c** Densely collagenous tissue replaces about 30 % of the mass, showing a few sparsely entrapped ductal epithelial components (H&E stain, ×40)

**Fig. 6:**
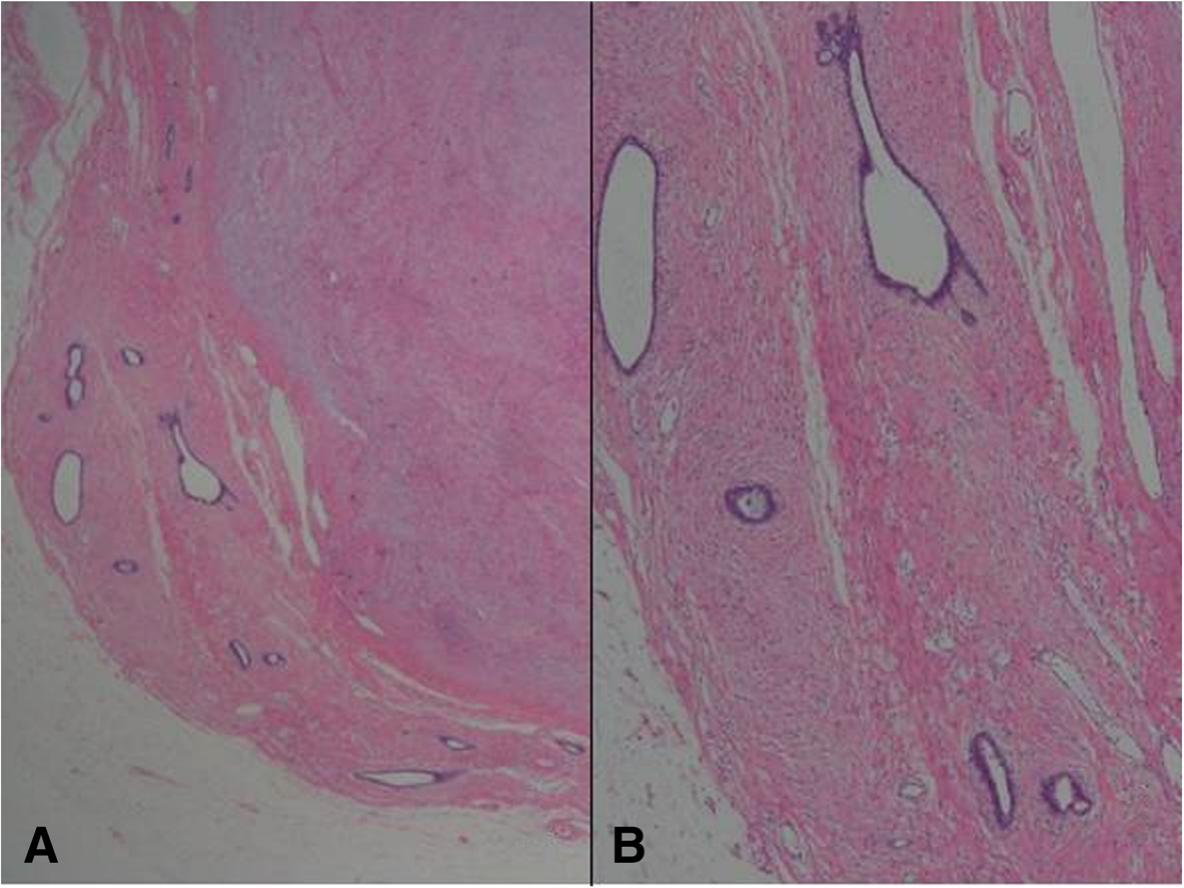
A peripheral region with fibroadenomatous remnants. **a** Round to ovoid ductal components are embedded in the myxoid cellular stroma (H&E stain, ×12.5). **b** The fibroadenomatous remnant shows still benign epithelial and mesenchymal components (H&E stain, ×40)

**Fig. 7:**
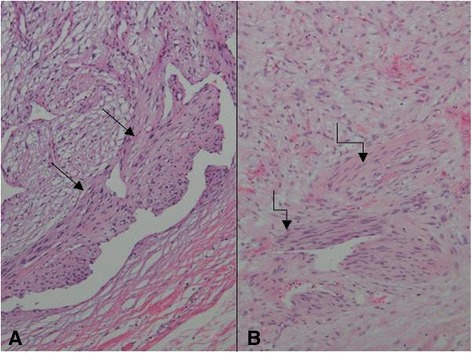
Intratumoral small vessels showing the transition to the tumor cells. **a** A peripheral venule-like vessel reveals intraluminally medial cell proliferation (arrows) (H&E stain, ×40). **b** Another tumor vessel is irregularly thickened by medial cell proliferation (curved arrows) of pericytic smooth muscle cells, which merge with the adjacent tumor cells (H&E stain, ×100)

**Fig. 8:**
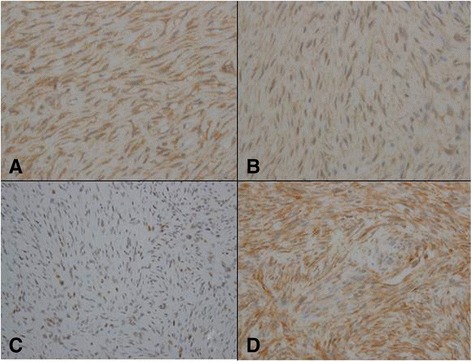
Immunohistochemical staining profiles favoring a myofibroblastic cell origin. **a** Diffuse fibronectin-immunoreactivity is found in the tumor cell cytoplasms (Diaminobenzidine, ×400). **b** Most tumor cell cytoplasms are immunoreactive for α-smooth muscle actin (Diaminobenzidine, ×400). **c** There is a diffuse nuclear immunopositivity for calponin, a myofibroblastic differentiation marker (Diaminobenzidine, ×200). **d** Vimentin, a general mesenchymal marker is immunostained strongly in most tumor cells (Diaminobenzidine, ×400)

A 61-year-old woman visited a breast clinic at our hospital, because she palpated a left breast mass first 8 months ago and felt that it has grown gradually in size from that time on. She had no family history of breast cancer and her physical examination revealed no axillary lymph node enlargement. Her mammography revealed a 3.5 cm-sized well-defined nodular mass with high density at 11 o’clock position of left breast, that was radiologically diagnosed as fibroadenoma (Fig. 1). She underwent a core needle biopsy initially under the impression of fibroadenoma, but the mass was diagnosed as being suggestive of myofibroblastoma at that time. Then, the mass was excised completely by a lumpectomy, which grossly revealed a slightly lobulated and ovoid nodular lesion, measuring 5×3×3 cm, and whitish yellow, solid, homogeneous, rubbery, and myxoid cut surface. Microscopic examination disclosed predominantly spindle cells with whorling or fascicular arrangement, which had mildly pleomorphic fusiform nuclei, moderate amount of eosinophilic fibrillary cytoplasm, and indistinct cytoplasmic borders (Fig. 2). Mitotic activity averaged 5–10 per 10 high-power fields (HPFs) but was found focally increased up to 16/10HPFs (Fig. 3). The nodular mass showed the hemorrhagic necrosis in about 5 % of entire tumor sections and a few foci of pseudocystic degeneration (Fig. 4). Background stroma was variably fibromyxoid according to the cellularity, ranging from being myxoid or loosely collagenous in the cellular areas to densely collagenous in the hypocellular or acellular areas (Fig. 5). Densely collagenous area amounted up to about 30 % and showed a few scattered ductal epithelial remnants of collapsed-linear or abortively oval shape. The peripheral portion of the mass also showed the ductal epithelial components embedded in the myxoid stroma, that suggests an ancient fibroadenomatous lesion (Fig. 6). Intriguingly, some intratumoral vessels revealed irregularly thickened walls with proliferating medial muscle cells that merge with adjacent intrinsic spindled tumor cells (Fig. 7). There were no areas of highly pleomorphic tumor cells like those of malignant fibrous histiocytoma. The tumor was relatively well-encapsulated, but focally showed the pushing-type extension outside the capsule. Immunohistochemical staining results of the tumor cells were diffusely positive for fibronectin, α-smooth muscle actin, calponin, and vimentin (Fig. 8). Immunoreactivities for CD34, desmin, pancytokeratin, bcl-2, estrogen receptor, and progesterone receptor were negative. The pericytes or medial muscle cells of the small venules were also immunoreactive for smooth muscle actin (SMA) and calponin, but not for desmin. All those light microscopic findings and immunohistochemical data suggested a myofibroblastic sarcoma of probably pericytic or vascular medial cell origin, with the background of ancient fibroadenomatous lesion. She got no additional chemotherapy or therapeutic radiation after lumpectomy. Follow-up data for 2 years after the lumpectomy revealed no evidence of local recurrence or distant metastasis on the mammogram, ultrasonography, Positron Emission Tomography, Magnetic Resonance Imaging of the liver, and abdominal Computed Tomography. Myofibroblasts are generally best identified in the granulation tissue but also might be originated from vascular smooth muscle cells or pericytes [9] or even from epithelial cells via metaplasia [10]. It has been a matter of considerable controversy whether or not a true myofibroblast can be neoplastic, because myofibroblasts can be found in the reactive conditions and benign neoplasms such as nodular fasciitis and fibromatosis. However, myofibroblastoma of the breast has been known as a distinctive benign mesenchymal tumor, since a study on 16 cases at 1987 showed that the myofibroblastoma cells are ultrastructurally different from fibroblasts, smooth muscle cells, or myoepithelial cells and microscopically characterized by uniformly slender and bipolar spindle cells haphazardly arranged in fascicular clusters with hyalinized fibrosis [11]. Thereafter, malignant tumors of myofibroblasts or those with myofibroblastic differentiation have been diagnosed in the breast by the presence of more aggressive histologic findings such as frequent mitotic counts, infiltrative growth pattern, and necrosis compared to myofibroblastomas. They are characterized clinically by variably malignant behavior ranging from recurrence to diffuse pleuropulmonary metastasis [1–7]. Myofibroblastic sarcoma or myofibrosarcoma (MFS) has been generally known to be identified best by their characteristic ultrastructural findings such as fibronectin extracellular fibrils and their fibronexus junctions, but it has been recently reported that the fibronectin can be demonstrated consistently by an immunohistochemical staining result alone [8]. Therefore, the present case could be diagnosed as MFS showing myofibroblastic differentiation with diffuse immunoreactivity for fibronectin. About 10 cases of mammary myofibroblastic sarcoma have been reported in the worldwide literature to the present, including the present case. The clinicopathologic features of only 8 cases among them are summarized for comparison in Table 1. Male to female ratio was 2:8 and the duration between disease onset and its diagnosis ranged from 1 week to 8 months. Immunohistochmical demonstration for myofibroblasts was done best by immunopositivities for SMA(100 %), vimentin(100 %), fibronectin(100 %), calponin(100 %), and bcl-2(50 %). Desmin, CD34, type IV collagen, laminin, h-caldesmon, and S100 protein were not immunoreactive in most cases. Four out of 8 cases had metastasis or recurrence, with 1 metastatic and 1 recurrent case being alive. Six cases (case 1–4, 6, 7) underwent ultrastructural studies, that usually demonstrated their myofibroblastic tumor cells with fibronectin extracellular fibrils and fibronexus junctions. However, any case report for mammary MFS have not reported or commented the background lesions such as fibroadenoma as like the present case. Also, any suggested cell origin of MFS has not been discussed yet within the tumor tissue in a worldwide literature. MFS is generally graded by two-tiered system as low and high-grades rather than low, intermediate, and high grades by mitotic count, nuclear pleomorphism, and necrosis. The former two grades of MFS share to a degree histopathologic findings of low-grade fascicular spindle cell neoplasm, whereas high-grade MFS show distinctively marked cellular pleomorphism mimicking MFH [8]. Therefore, our case could be diagnosed cytologically as low-grade myofibrosarcoma (LG-MFS). The most representative myofibroblastic tumors of the breast are myofibroblastoma and LG-MFS. LG-MFS is a predominantly spindle cell neoplasm that could show frequent mitoses and focal necrosis, whereas benign myofibroblastic tumors including mammary-type myofibroblastoma don’t reveal the two. In addition, LG-MFS of the breast may have a wide range of differential diagnoses such as leiomyosarcoma, fibrosarcoma, and cystosarcoma phyllodes with fibrosarcoma-like overgrowth, but the most important differential diagnosis in the present case is the last one. Both lesions can show epithelial entrapment within the tumor, but there are obvious differences in morphological, immunohistochemical, and ultrastructural study results for the sarcoma cell nature. The overgrown fibrosarcoma-like lesion arising in cystosarcoma phyllodes generally show cleft-like compressed epithelial components and the fibroblastic features rather than myofibroblasts by the immunohistochemical and ultrastructural studies. In contrast, the present tumor revealed multifocally residual linear or round ductal epithelial remnants mainly in the hyalinized collagenous stromal backgrounds, mimicking histologically ancient fibroadenoma, and immunohistochemically diffuse fibronectin reactivity in the tumor cells. With regard to the sarcoma component alone, the most important differential diagnoses may include fibrosarcoma, leiomyosarcoma, and inflammatory myofibroblastic tumor. Fibrosarcoma is composed of malignant spindle cells showing fibroblastic differentiation which is different from myofibroblasts by no immunohistochemical evidence of fibronectin, SMA, calponin. Leiomyosarcoma is also a malignant spindle cell tumor with smooth muscle features that are characterized by the presence of immunoreactivities for H-caldesmon, desmin, and occasionally keratin but no evidence of immunoreactivity for fibronectin. Thus, leiomyosarcoma and MFS can be differentiated by those immunohistochemical staining results, in spite of common immunoreactivity for SMA. Finally, inflammatory myofibroblastic tumor (IMT) can be differentiated from MFS by its microscopic findings such as diffuse inflammatory infiltrates of lymphoplasma cells, eosinophils, and histiocytes and no evidence of nuclear pleomorphism or atypical mitotic figures. Also, IMT shows a relatively frequent immunoreactivity for ALK, an important diagnostic marker for differential diagnosis with MFS. With the diffuse immunoreactivity for fibronectin, MFS can be differentiated from leiomyosarcoma and malignant fibrous histiocytoma, because the latter two use type IV collagen to connect to the extracellular matrix [6]. Pericytes were discovered first by Charles Rouget and are generally known as mural cells or vascular smooth muscle cells because of their contractile fibers [12]. They are quite abundant on small venules and arteries but are rather sparse on capillaries. They exihibit a number of characteristics consistent with muscle cell activity and thus can express smooth muscle actin. However, it is still not clear whether pericytes are smooth muscle cells or cells with smooth muscle cell characteristics that can turn into smooth muscle cells, which could suggest that pericytes and smooth muscle cells represent phenotypic variants of the same lineage, or even have a distinct progenitor [12]. Therefore, pericytes share the smooth muscle actin with myofibroblast and thus have been considered a possible origin of myofibroblastic neoplasm. The present case showed some intratumoral vessels of small venule size to be irregularly thickened by mural cell proliferation of probably pericytic origin, which merged with the myofibroblastic sarcoma cells (Fig. 7). Another putative cell origin could be the uncommitted vimentin+/CD34+ fibroblast of mammary stroma, because it is thought to be capable of multidirectional differentiation into fibroblastic, myofibroblastic, leiomyomatous, adipocytic, osseous, and cartilaginous lines [13]. However, it is less likely candidate of tumor cell origin in this case, because the tumor cells don’t’ express CD34-immunoreactivity and no evidence of transition to mammary stromal cells.

**Table 1:** Case Summary and Comparison of 8 Mammary Myofibrosarcomas in the Worldwide Literature (1997-2015)

Case No.	Clinical Findings	Pathologic Findings	Immunohistochemical Findings	Therapy and F/U data
Sex/age(Pre.y)	Site, Size, DUR	Grade, Necrosis, Mitosis	Positive/Negative	
1. F/55(1997)	UOQ, 2- > 8 cm, 1 W	High Gr, +, 10–45/10HPFs	VM,SMA,FN;+/DSM,LMN,COLIV;−	Mastectomy with LA. Died 11 M with meta.
2. M/60(1999)	UOQ, 2.5 cm, 1 M	Low Gr, +, 10/10HPFs	VM,SMA;+/DSM,LMN,S100,CD34;−	Mastectomy with LA. Alive 10Y with recurrence
3. F/59(1999)	NC, 2.3 cm, NC	Low Gr, -, 6–15/10HPFs	VM,SMA,FN;+/DSM,LMN,S100,CD34;−	Mastectomy with LRT. NED 20 M
4. F/72(2001)	NC, 3.4 cm, NC	Low Gr, +, 2/10HPFs	SMA,MSA,DSM:+/Cytokeratin:−	Mastectomy. Died 12 M with meta.
5. F/51(2003)	4Qs, 22 cm, 3 M	High Gr, + 8–35/10HPFs	VM,SMA;+/S100,DSM,h-CAL,PCK;−	Mastectomy with CRT. Alive 24 M
6. F/46(2005)	UOQ, 2 cm, 6 M	Low Gr, -, NC	VM,SMA,FN;+/PCK,COLIV;−	Mastectomy. NED 12 M
7. F/81(2011)	NC, 4.2 cm, 2 W	Low Gr, NC, many/10HPFs	VM,SMA,bcl-2;+/DSM,S100,c-kit,CD34;−	Mastectomy with RT. Alive 16 M with meta.
8^a^. F/61(2015)	UOQ, 3–> 5 cm, 8 M	Low Gr, +, 5–16/10HPFs	VM,SMA,FN,calponin;+/DSM,CD34,p63,bcl-2;−	Lumpectomy. NED 18 M

^a^: the present case, *Pre.y* presented year, *DUR* duration, *F/U* follow-up, *UOQ* upper outer quadrant, *W* week, *Gr* grade, *HPF* high-power field, *VM* vimentin, *SMA* smooth muscle actin, *FN* fibronectin, *DSM* desmin, *LMN* laminin, *COLIV* collagen type IV, *LA* lypmphadnectomy, *M* month, *meta.* metastasis, *Qs* quadrants, *Y* year, *NC* no comment, *LRT* local radiation therapy, *NED* no evidence of disease, *h-CAL* h-caldesmon, *PCK* pancytokeratin, *CRT* chemoradiation therapy, *RT* radiation therapy

## Conclusions

A myofibroblastic sarcoma of the breast could develop spontaneously by sarcomatous transformation of vascular pericytes or medial muscle cells in a long-standing fibroadenoma. This is the first case report of low-grade myofibroblastic sarcoma showing a tumor cell origin and a benign background lesion in the worldwide literature.

## Consent

Written informed consent was obtained from the patient for publication of this case report and any accompanying images. A copy of the written consent is available for review by the Editor-in-Chief of this journal.

## Abbreviations

ALK: Anaplastic lymphoma kinase
CD34: Cluster designation 34
h-caldesmon: High molecular weight isoform of caldesmon
